# A Retrospective Interventional Study Examining Whether Successful Replacement Therapy After a Confirmed Vitamin D Deficiency Correlates with Improved Disease-Free Survival in the Curative Intent Treatment of HER2+ Breast Cancer

**DOI:** 10.3390/nu18081253

**Published:** 2026-04-16

**Authors:** Eugene R. Ahn, Nandhini Iyer, Samuel B. Cothran

**Affiliations:** 1 Cardinal Bernardin Cancer Center, Loyola University, Maywood, IL 60153, USA; 2 Loyola University Health System, MacNeal Hospital, Berwyn, IL 60402, USA; nandhini.iyer1998@gmail.com; 3 Walter Reed National Military Medical Center, Bethesda, MD 20889, USA; samuel.b.cothran.mil@health.mil

**Keywords:** HER2+ breast cancer, vitamin D, vitamin D2, vitamin D3, serum 25-hydroxyvitamin (D25), *TP53* mutation

## Abstract

Background: Vitamin D is a secosteroid that exerts immunomodulatory and anti-proliferative effects through the vitamin D receptor (VDR). Because HER2-targeted therapies substantially improve prognosis in HER2-positive breast cancer and introduces a new mechanism of immunotherapy, we hypothesized that successful correction of vitamin D deficiency would be associated with improved disease-free survival (DFS) in patients treated with curative intent. Methods: We performed a retrospective interventional cohort study of patients with early-stage HER2-positive breast cancer treated at Cancer Treatment Centers of America Midwestern Regional Medical Center from 2008 to 2014. Eligible patients had baseline vitamin D deficiency (25-hydroxyvitamin D or D25 < 30 ng/mL), received trastuzumab-based therapy, and had ≥12 months follow-up. Patients received vitamin D3 supplementation (typically 2000–10,000 IU/day) with doses adjusted based on D25 level follow-up. Responders were defined as having achieved a mean D25 ≥ 30 ng/mL during the first year; non-responders remained <30 ng/mL DFS was analyzed using Kaplan–Meier and Cox models. Results: Of 196 patients, 129 (65.8%) were vitamin D-deficient at baseline. Among these, 76 (60.3%) achieved repletion while 50 (39.7%) remained deficient. Three did not have D25 follow-up obtained. Thirty-one DFS events occurred but no deaths. Responders demonstrated numerically improved outcomes (3-year DFS 90% vs. 85%). Non-responders had a 1.7-fold higher hazard of recurrence, and those who achieved the highest D25 levels (>50 ng/mL) had the most favorable DFS trends, suggesting a dose response. Conclusions: Failure to correct a vitamin D deficiency was associated with a 1.7-fold higher recurrence risk, although the relationship did not achieve statistical significance. A similar effect size was reported in another retrospective cohort of HER2-positive breast cancer that did achieve statistical significance, and a doubling of pCR rates was seen in two recently completed RCTs in 2025, with benefits particularly seen in the triple-negative and HER2-positive subtypes. Prospective trials evaluating optimized vitamin D repletion in HER2-positive breast cancer are warranted.

## 1. Introduction

Breast cancer is the most common cancer among women in the United States, with an estimated 321,910 new invasive cases and 42,140 deaths projected in 2026, accounting for nearly one-third of all new cancer diagnoses in women [1]. Breast cancer is a biologically and clinically heterogeneous disease, encompassing multiple tumor subtypes with distinct cellular and molecular features that contribute to variability in prognosis and optimal treatment options [2].

Vitamin D is a fat-soluble secosteroid that is primarily synthesized in the skin following ultraviolet light exposure, making the term “vitamin” (normally defined as a trace dietary constituent) technically a misnomer [3], although it can also be obtained from dietary and/or supplemental sources. Vitamin D serves as a precursor to the active steroid hormone 1,25-dihydroxyvitamin D (calcitriol) which subsequently binds to vitamin D receptor (VDR) and retinoic X receptor heterodimeric complex and regulates the expression of >1000 genes [4].

**Figure 1:**
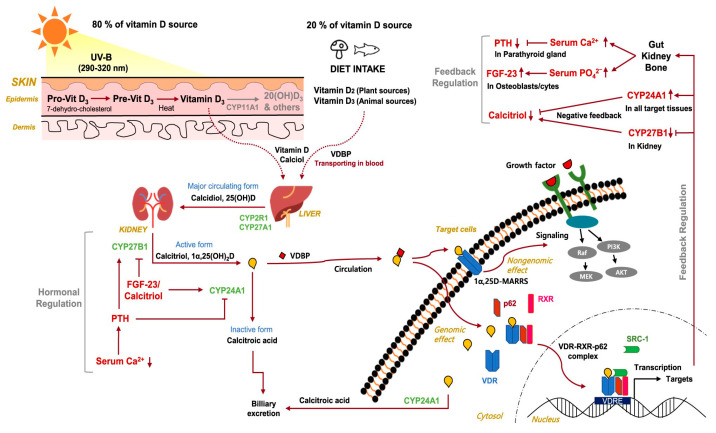
An overview of vitamin D metabolism. Vitamin D as an endogenous secosteroid with many effects on human health and cancer outcomes primarily through its interactions with vitamin D receptor (VDR) [6]. Pre-vitamin D3 is primarily converted to D3 in the skin with UV-B exposure, then hydroxylated by CYP2R1 or 25-hydroxylase in the liver to 25(OH)D, and lastly CYP27B1 or 1-alpha hydroxylase in the renal tubules to calcitriol or 1,25(OH)D. Vitamin D metabolites are stabilized in the serum by Vitamin D Binding Protein (VDBP). The genomic effects of 1,25(OH)D occur through binding to VDR-RXR complexes which subsequently interact with Vitamin D Responsive Elements (VDRE) on DNA. Additionally, non-genomic effects can be mediated through interaction of 1,25(OH)D with membrane associated rapid response steroid (MARRS) receptor.

While rickets and the classic bony abnormalities (frontal bossing, bow leggedness) associated with a vitamin D deficiency were first reported during Europe’s industrial revolution in the mid-17th century [5], vitamin D is now recognized for its many extraskeletal functions, including but not limited to modulation of immune responses, attenuation of inflammatory pathways, and regulation of cellular proliferation and differentiation (Figure 1) [6].

Vitamin D consists of a family of two compounds, vegetable and fungal ergocalciferol (D2) and animal cholecalciferol (D3), although D3 produced by lichen is commercially available. Serum 25-hydroxyvitamin D (D25) assays typically measure both D2 and D3 variants. Normal vitamin D25 levels do not always translate to physiological sufficiency most commonly in the setting of renal failure, with rare exceptions including individuals with vitamin D-dependent rickets type IA (VDDR-1A caused by inactivating variant of *CYP27B1* or 1-alpha hydroxylase) or hereditary vitamin D-resistant rickets (HVDRR caused by autosomal recessive mutations in *VDR*). Also, low vitamin D25 levels do not always translate to functional deficiency, for example, in individuals with genetic polymorphisms in the vitamin D-binding protein (*VDBP*) particularly seen in African Americans. Lastly, patients with vitamin D-dependent rickets type IB (VDDR-IB caused by mutations in *CYP2R1* or 25-hydroxylase) may not benefit from D2/D3 supplementation.

Retrospective case–control studies focused on the association of D25, currently the best practical proxy for vitamin D status, with breast cancer subtype risk [7,8], have demonstrated that a vitamin D deficiency is associated with a greater risk of higher-grade tumors and ER negative subtypes. In a meta-analysis limited to prospective cohorts [9], no statistically significant associations were found, but there was a suggestion of association with triple-negative breast cancer and distant metastases. The research connecting vitamin D deficiency with higher risk of overall cancer mortality is much stronger than breast cancer incidence [10,11].

Randomized controlled trials (RCTs) on vitamin D supplementation focused on breast cancer-specific outcomes are limited but all three have been published 2024–2025 and all were focused on neoadjuvant chemotherapy. Two of the three randomized clinical trials were positive and showed a doubling of the odds of pathological complete response rate (pCR) [12,13]. The third negative study enrolled 76 patients into 4 arms, resulting in a heavily underpowered study [14]. A major weakness of all three studies is that all subtypes of breast cancer were enrolled, including ER+ subtypes, and, of note, pCR has been shown to be relevant to long term prognosis in triple-negative breast cancer and ER-HER2+ breast cancer [15,16] but not so in ER+ breast cancer.

With the emergence of HER2-targeted monoclonal antibodies for the treatment of HER2+ breast cancer, it has been previously queried whether vitamin D intake might be clinically relevant in HER2+ breast cancer [17]. In this retrospective analysis of all patients receiving curative intent trastuzumab (+/− pertuzumab) at a single academic institution, there was a statistically significant improvement in disease-free survival (DFS) after multivariate analysis in those taking vitamin D supplementation (HR 0.36 *p* = 0.03). A weakness of the study was that D25 measurements at baseline and follow-up were not available.

At Cancer Treatment Centers of America, Midwestern Regional Medical Center (CTCA MRMC), it was standard of care to routinely screen all new patients with a D25 and follow serial measurements to assure adequate replacement was provided. Leveraging this unique clinical practice, we examined whether successful correction of vitamin D deficiency was associated with improved disease-free survival among patients with HER2-positive breast cancer.

## 2. Materials and Methods

### 2.1. Study Design and Population

We conducted a retrospective interventional cohort study of patients with HER2+ breast cancer treated at CTCA MRMC (located in the north suburbs of Chicago, i.e., Zion IL) between 2008 and 2014. The study was approved by the Institutional Review Board of CTCA MRMC.

Patients were selected without risk of bias by beginning with a list of all patients treated with trastuzumab +/− pertuzumab at the institution. Patients were included for final analysis if they had a diagnosis of early-stage HER2+ breast cancer, and continued care at CTCA for a minimum of 12 months from intake and had a baseline vitamin D deficiency, i.e., serum D25 < 30 ng/mL. The inclusion criteria were predefined in the IRB protocol before data extraction was initiated.

Histological confirmation including HER2 IHC status (3 + IHC) was done in all cases with core biopsies of the primary tumor (and if applicable, additional lymph node biopsy) before start of neoadjuvant chemotherapy or surgery, and for those with 2 + IHC equivocal results, HER2 FISH with a ratio of HER2/CEP17 greater than 2.0 would result in classification of HER2-positive status. ER or PR positivity by IHC was defined as ≥1%.

The study hypothesis was that successful vitamin D supplementation would be associated with an improved disease-free survival (DFS) using Kaplan–Meier methods. A secondary aim was to see if there was any evidence that targeting higher levels, i.e., >50 ng/mL, might have an additional benefit and to see if there was a “dose response” with higher tiers of D25 levels achieved.

### 2.2. Exposure Assessment and Vitamin D Responder Classification

Baseline vitamin D status was determined from an intake visit D25 (standard of care for all patients seen at CTCA MRMC prior to initial consultation). Vitamin D deficiency was defined as a baseline D25 < 30 ng/mL. Patients who were vitamin D-deficient at baseline received vitamin D supplementation (most commonly D3, but if patient preferred D2 it was allowed, and generally USP certified brands were preferred) as part of routine clinical care through consultation with a naturopath (also standard of care) and the medical oncology team. Although a formal protocol for replacement was not defined by the institution, starting replacement doses ranged from 2000 to 10,000 IU/d D3 and were adjusted accordingly based on subsequent D25 measurements.

The D25 assay was done in-house utilizing the Roche Cobas 6000 vitamin D total assay, an automated chemiluminescent immunoassay. The system employs VDBP labeled with a ruthenium complex as capture protein to bind 25-hydroxyvitamin D3 and D2. Cross-reactivity to 24,25-dihydroxyvitamin D is blocked by a specific monoclonal antibody.

The primary exposure of interest was response to vitamin D supplementation. Among patients with baseline vitamin D deficiency, a response was determined from the mean D25 concentration measured after the intake assessment through the end of the first year (to mirror the typical duration of HER2-targeted therapy). Patients were classified as responders if the mean follow-up D25 level was ≥30 ng/mL and as non-responders if the mean D25 remained <30 ng/mL. Responders were further stratified into low (30–40 ng/mL), medium (40–50 ng/mL), and high (>50 ng/mL) responder categories.

### 2.3. Outcomes

The primary outcome was disease-free survival (DFS). DFS was defined as the interval from the date of initiation of neoadjuvant therapy or definitive surgery, whichever occurred first, to the earliest documented disease recurrence, development of metastatic disease, or death from any cause. Patients without disease progression were censored at the date of last clinical follow-up.

### 2.4. Covariates

Clinical and pathologic covariates of interest included age at diagnosis, body mass index (BMI), estrogen receptor (ER) status, progesterone receptor (PR) status, tumor size, number of metastatic lymph nodes, presence of lymphovascular invasion, type of chemotherapy (neoadjuvant vs. adjuvant), use of pertuzumab, type of definitive surgery, and receipt of radiation therapy.

### 2.5. Statistical Analysis

Baseline patient characteristics were summarized using frequencies and percentages for categorical variables and appropriate measures of central tendency for continuous variables. Differences across groups were assessed using standard comparative statistical methods, as appropriate.

DFS was estimated using the Kaplan–Meier method, and survival curves were compared using the log-rank test. Univariate Cox proportional hazards regression models were first used to assess the association between individual covariates and DFS. Variables demonstrating clinical or statistical relevance were subsequently included in multivariable Cox proportional hazards models to evaluate independent associations after adjustment for potential confounders. Effect estimates were reported as hazard ratios (HRs) with corresponding 95% confidence intervals (CIs).

The proportional hazards assumption was evaluated using both graphical methods, including log-minus-log plots for categorical variables, and statistical testing through extended Cox models incorporating time-dependent covariates for continuous variables. A two-sided *p*-value ≤ 0.05 was considered statistically significant. All analyses were performed using IBM SPSS Statistics, version 28.0 (IBM Corp., Armonk, NY, USA).

## 3. Results

### 3.1. Study Cohort and Baseline Characteristics

A total of 196 patients met the initial eligibility criteria and were then subcategorized into vitamin D sufficiency or deficiency status at baseline. Baseline demographic and clinicopathologic characteristics are summarized in Table 1. Of the 196 patients eligible for analysis, 129 patients (65.8%) had deficient D25 levels (<30 ng/mL), while 67 patients (34.2%) had sufficient levels (≥30 ng/mL).

Nearly half of the patients had a body mass index (BMI) > 30 kg/m^2^ (44.9%). Most patients were White (66.3%), followed by African American (21.4%) and Hispanic (7.7%). Most tumors measured <2 cm (64.1%), and 55.7% of patients had no metastatic lymph node involvement. Lymphovascular invasion was present in 36.2% of cases.

Hormone receptor positivity was common, with estrogen receptor (ER) positivity in 70.5% and progesterone receptor (PR) positivity in 53.4% of patients. Approximately half of the cohort received neoadjuvant chemotherapy (49.5%), while the rest received adjuvant chemotherapy (50.5%). Definitive surgery consisted predominantly of mastectomy (56.9%), and most patients received adjuvant radiation therapy (73.5%). Pertuzumab was used in 50% of patients.

### 3.2. Baseline Vitamin D Status and Response to Supplementation

Among the 129 patients who were vitamin D-deficient at baseline, 76 patients (60.3%) achieved adequate vitamin D repletion (defined by mean D25 > 30 ng/mL in the first year of follow-up) and were classified as responders, while 50 patients (39.7%) remained below the target threshold and were classified as non-responders. Vitamin D response status could not be determined for three patients due to incomplete follow-up laboratory data and were not included for analysis. Among the 76 responders, 27 patients were classified as low responders (mean follow-up D25 level 30–40 ng/mL), 31 medium responders (40–50 ng/mL), and 18 high responders (>50 ng/mL).

### 3.3. Disease-Free Survival in the Overall Cohort

**Figure 2:**
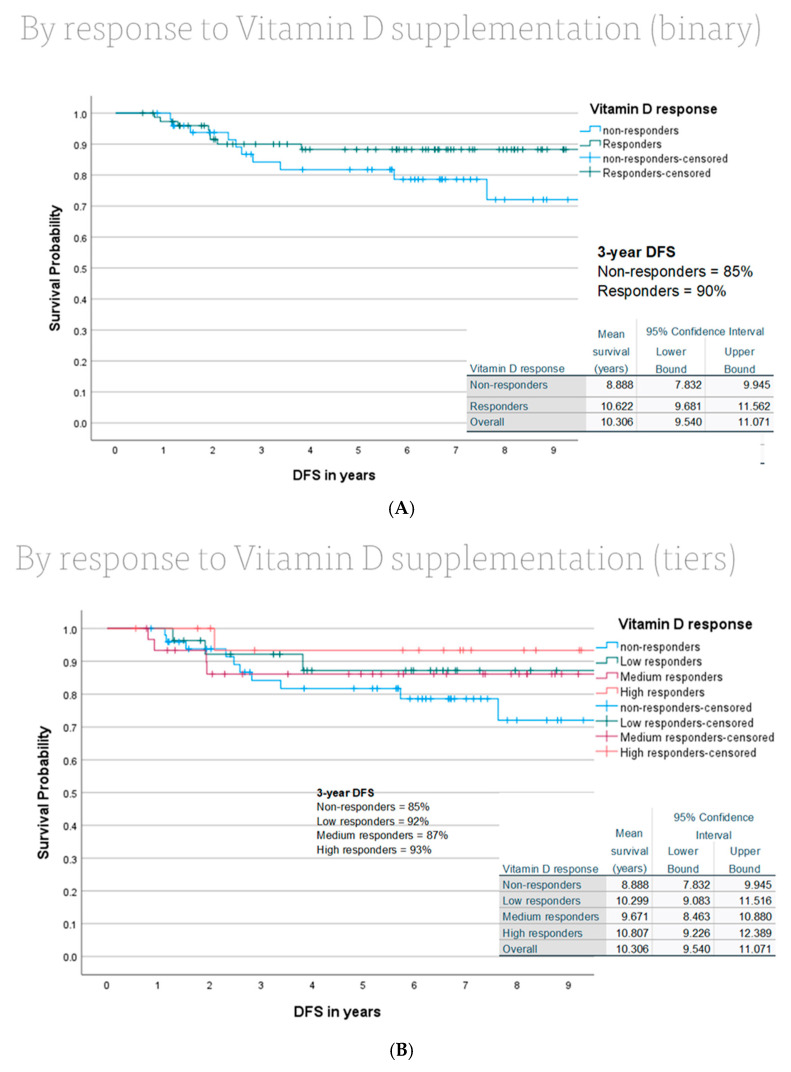
(**A**) Kaplan–Meier DFS curves analyzed by vitamin D responders versus non-responders. (**B**) Kaplan–Meier DFS curves by tiers of vitamin D response versus non-responders, specifically low responders (30–40 ng/mL), medium responders (40–50 ng/mL) and high responders (>50 ng/mL).

During follow-up, 31 of 196 patients (15.8%) experienced a disease-free survival (DFS) event. No deaths were recorded, so overall survival analysis was not possible, and in essence, DFS in this cohort would be accurately reframed as recurrence-free survival (RFS). Owing to the relatively low number of disease recurrence events, the median DFS was not reached. The mean DFS for the overall cohort was 10.2 years (95% confidence interval [CI], 9.58–10.83). The estimated 3-year DFS rate for the entire cohort was 88%, with non-responders having an 85% 3 yr DFS and responders having a 90% 3 yr DFS, as shown in Figure 2A.

In Figure 2B, an apparent “dose response” separation of the curves can be seen for those who were non-responders, and low, medium, and high responders to vitamin D replacement therapy, with the best outcomes associated with those who achieved mean D25 > 50 ng/mL.

### 3.4. Cox Proportional Hazards Analysis

Univariate and multivariable Cox proportional hazards regression analyses were performed to identify factors associated with DFS. See Table 2 for Univariate Cox Proportional Hazard Analysis. In univariate analyses, BMI, lymphovascular invasion, treatment sequence, and pertuzumab use were significantly associated with DFS. Patients with BMI ≥ 30 kg/m^2^ surprisingly demonstrated a lower hazard of disease progression compared with those with BMI < 30 kg/m^2^ (HR 0.41 *p* = 0.03). The presence of lymphovascular invasion was associated with a higher hazard of disease progression (HR 2.30, *p* = 0.04). Patients treated in the neoadjuvant setting versus adjuvant (only) exhibited a higher hazard of disease progression (HR 3.80 *p* = 0.001) as well as those who received pertuzumab (HR 2.50 *p* = 0.02), likely due to selection bias of more advanced stage presentations.

DFS did not differ significantly by age at diagnosis, i.e., ≥50 years versus < 50 (HR 1.1 *p* = 0.7), race, i.e., non-White subjects versus White subjects (HR 1.3, *p* = 0.3), tumor size, i.e., ≥2 cm vs. <2 cm (HR 1.3 *p* = 0.3), nodal status, i.e., ≥1 metastatic versus 0 lymph nodes (HR 1.4 *p* = 0.3), estrogen receptor status, i.e., positive versus negative (HR 0.79 *p* = 0.5), progesterone receptor status, i.e., positive versus negative (HR 0.81 *p* = 0.6), nor baseline vitamin D25 status, i.e., <30 ng/mL versus > 30 ng/mL (HR 0.76 *p* = 0.45).

Although a binary analysis of White vs. non-White ethnicity was not significant, African Americans continued to have the worst 3 yr DFS of 79%, compared to 89% for White subjects and 100% for Hispanics.

When using vitamin D responders as reference, vitamin D non-responders had a HR of 1.7 for worse DFS, although the *p*-value was not significant *p* = 0.23.

Multivariable Cox proportional hazards regression (Table 3) was subsequently performed to evaluate independent associations with DFS after adjustment for BMI, lymphovascular invasion, treatment sequence, and pertuzumab use. In the adjusted model, lymphovascular invasion remained independently associated with worse DFS (HR 2.3 *p* = 0.05). Treatment sequence also remained independently associated with DFS, with neoadjuvant therapy associated with a higher hazard of disease progression compared with adjuvant therapy (HR 4.0 *p* = 0.008). After adjustment, BMI ≥ 30 kg/m^2^ continued to demonstrate a sizable lower hazard of disease progression compared with BMI < 30 kg/m^2^ with HR 0.46; however, it lost its statistical significance (*p* = 0.09). Pertuzumab use was not independently associated with DFS (HR 1.20 *p* = 0.69).

## 4. Discussion

This is the first retrospective interventional cohort focused on women with HER2+ breast cancer with baseline and serial D25 measurements where all patients were given replacement therapy if deficient and doses were adjusted over time based on follow-up D25. The results suggest that not successfully fixing the vitamin D deficiency with appropriate vitamin D dosing could lead to a 70% higher risk of recurrence in patients with HER2+ breast cancer, although the confidence of this conclusion is weakened by the lack of statistical significance.

Because this cohort is not a retrospective observational cohort, but a retrospective interventional study (although not wholly accurate, we would call this a retrospective implementation study), the documentation of dose response (D25) correlation to improved DFS provides a fidelity component to this data that does not prove causation, but does provide a stronger clinical signal than a purely retrospective observational cohort that achieved statistical significance. Larger prospective “implementation studies” are warranted now for more definitive evidence.

In a previously reported single-institution observational cohort of patients treated with trastuzumab-based chemotherapy for localized HER2+ breast cancer, vitamin D intake (mean 10,472 IU/week) was associated with a HR of 0.36 (*p* = 0.03) after multivariate analysis [17]; however, a major weakness of the study was the lack of routine D25 intake measurements and follow-up, so analysis based on D25 levels achieved was not possible.

Another reason for publishing these findings are two recent randomized controlled trials showing a doubling of pCR in patients undergoing neoadjuvant chemotherapy for breast cancer [12,13], with benefit mainly seen in the HER2+ and triple-negative subtypes.

In the Omodei study (*n* = 80) [12], patients received 2000 IU/d of D3 versus placebo, and mean baseline D25 was 19.6 and 21.0 ng/mL in the vitamin D and placebo groups respectively, with confirmed difference in D25 levels at end of study 28.0 vs. 20.2 ng/mL. The pCR rate was 43% vs. 24% *p* = 0.04. When comparing those who achieved pCR versus those who did not, there was a higher prevalence of HER2+ and triple-negative subtypes (16% vs. 6%, and 40% vs. 18%, *p* = 0.02, respectively).

In the Özkurt 2025 study (*n* = 227) [13], patients were randomized to either D3 50,000 IU/week or no vitamin D supplementation. Similarly, patients were vitamin D-deficient at baseline 23 ng/mL in both arms, and 57% (average 71 ng/mL) achieved sufficiency in the intervention arm versus 18.4% in the control. The pCR rate was 24.3% versus 10.6% *p* = 0.012. In subset analysis, the pCR benefit was mainly seen in the HER2+ and triple-negative subtypes compared to ER+ subtypes (*p* = 0.016 and *p* = 0.004, respectively).

### 4.1. Limitations and Additional Strengths

Some of the limitations of this study include it being retrospective, single-institution, and likely underpowered. The underpowering due to low frequency of DFS events is likely multifactorial. First, it is unusual to have a HER2+ breast cancer cohort where nearly two-thirds of the tumors are <2.0 cm. Second, the majority of the HER2+ breast cancer cases were not of the ER-HER2+ subtype (70.5% were ER+). We still included this subset because, unlike the neoadjuvant studies above, our study period was not limited to neoadjuvant therapy and was inclusive of adjuvant therapy when patients were receiving antiestrogen therapy with trastuzumab +/− pertuzumab. However, ER+HER2+ breast cancer has a better prognosis than ER-HER2+ breast cancer and has outcomes approaching luminal tumors [18]. This underpowering increases the risk that these findings were by chance.

Other study weaknesses include the study definition of exposure and the requirement of follow-up of D25 levels in the subsequent year without specification of the intervals for testing D25 and without a more specified standardized replacement protocol based on BMI. For example, patients who were “responders” might have been generally more compliant with conventional therapy and had better prognosis as a result and “non-responders” might have had overlap with socioeconomic difficulties as a confounder, had less frequent D25 tests done and had less efficient dose adjustments over the first year. Lack of sufficient doses given to high BMI patients also likely underpowered the study. Vitamin D replacement treatment heterogeneity based on different clinician preferences might have created additional confounding variables.

Since we included both patients receiving neoadjuvant therapy and patients receiving adjuvant therapy, we did introduce more conventional treatment heterogeneity and our multivariate analysis did show that those who received neoadjuvant chemotherapy had a 4-fold worse prognosis (*p* = 0.008) due to selection of more advanced stages, and pertuzumab use nearly was significant (*p* = 0.07), as pertuzumab is indicated in patients with more advanced stages. These additional confounders also limit the generalizability of the findings.

Lastly, although none of the patients in this cohort had renal failure or were on dialysis, a formal assessment and categorization of renal insufficiency was not performed, which theoretically could have influenced results, since in such patients D25 levels might not have accurately reflected bioactive vitamin D due to impaired activation of D25 in the renal tubules.

A strength is that the patient population queried was more diverse and representative of a typical American population compared to those enrolled in previous RCTs, with a greater proportion of obese patients (44.9% > 30 kg/m^2^) and ethnic minorities, including African Americans (21.4%) and Hispanics (7.7%).

### 4.2. On the Role of BMI

A surprising finding in our cohort was that a high BMI emerged as a favorable prognostic factor. Although not statistically significant in multivariate analysis, the HR for disease recurrence for high BMI patients > 30 kg/m^2^ was 0.46, which is contrary to what was expected based on previous publications associating high BMI with worse outcomes in curative intent treatment of ER-HER2+ breast cancer [19,20] in the post-trastuzumab era, and in the pre-trastuzumab era [21].

It is our hypothesis that this reversal in the fortunes of high BMI patients could represent a clinical signal that a high BMI in part is a negative prognostic factor in HER2+ breast cancer through the mechanism of serving as a “vitamin D physiological trap” [22,23,24]. Vitamin 1,25(OH)D has also been shown to inhibit leptin and IL-6 production by adipocytes [25,26]. It is possible that a high BMI in this cohort selected out patients who were more likely to benefit from a D25-guided replacement therapy approach where patients required doses as high as 25,000 IU/d to achieve normal levels.

### 4.3. Review of Putative Mechanisms of Vitamin D Bioactivity in HER2+ Breast Cancer

Although our study did not query molecular insights into the reason for potential biological activity, it is important to review the large evidence base supporting the biological plausibility of such activity.

Vitamin D is a steroid prohormone and requires sequential hydroxylation to become biologically active, with both renal and extrarenal tissues, including breast epithelium, capable of activating circulating 25-hydroxyvitamin D to 1,25-hydroxyvitamin D or calcitriol [27]. Calcitriol signaling through the vitamin D receptor (VDR) influences multiple cancer-relevant pathways, including the inhibition of cellular proliferation via cell-cycle arrest, the induction of apoptosis through the modulation of *Bcl-2* family proteins, the inhibition of matrix metalloproteinases involved in tumor progression, the downregulation of *COX-2* expression and associated inflammation, and the suppression of additional invasive and angiogenic processes [6,27]. Recent evidence shows that vitamin D stimulation of intestinal *VDR* induces an improved microbiome and subsequent anti-tumor immunity in mouse models [28].

Vitamin D has been shown to enhance NK cell activity, providing a mechanism by which vitamin D might be particularly beneficial in HER2+ breast cancer treated in the post-trastuzumab era [29]. Preclinical models looking at another monoclonal antibody rituximab for lymphoma identified that maximal NK cell activity was observed at 65 ng/mL, supporting our findings that high responders, D25 levels > 50 ng/mL, had the best nominal DFS. Preclinical studies suggest vitamin D analogs may inhibit HER2-associated downstream signaling pathways, including the *ERK* and *PI3K*/*AKT* cascades [30,31]

Another potential mechanism for the biological activity of vitamin D in HER2+ breast cancer (and ER- breast cancer) is the known cross-talk between *TP53* and *VDR* [32]. HER2+ breast cancer has a high prevalence of *TP53* mutations, 55–75% [33], and patients with germline *TP53* mutations are more likely to develop HER2+ breast cancer (67%) versus other subtypes [34]. In a subset analysis of *TP53* mutated gastrointestinal cancers (using p53 immunoreactivity as a proxy) in the AMATERASU study [35], vitamin D supplementation led to 81% 5-year relapse free survival versus 31% in the placebo group (HR 0.27 95% CI 0.11–0.61), suggesting *TP53* mutations might be an important biomarker for cancers more likely to benefit from vitamin D supplementation [36].

## 5. Conclusions

The inefficiency of vitamin D repletion (60.3% success rate) in our cohort emphasizes that a fixed dose replacement strategy, even with adjustments based on serial D25 measurements, does not reliably translate into adequate repletion in real-world practice, particularly for high BMI patients [37,38,39]. To resolve this underpowering issue in future prospective studies, it would be prudent for future clinical trials to focus on interventions utilizing scientifically proven dosing formulas based on patient weight and baseline D25 [40].

As of 2026, it is currently not in the ASCO guidelines to screen and address a vitamin D deficiency in patients newly diagnosed with cancer unless they are about to start a bone weakening agent [41]; however, it is NCCN guideline-supported to screen for and treat any nutritional deficiencies concurrent with cancer treatment [42].

A total of 70.3% of Americans have a vitamin D deficiency (<30 ng/mL) [43]. Of note, our study cohort had a similar prevalence of 65.8%. Given the high pretest odds, particularly for African Americans (94.5%) and Hispanics (86.5%) and obese individuals (80.1%) [43], and that vitamin D deficiency is commonly associated with quality-of-life issues, particularly fatigue [44,45,46], routine screening and replacement therapy should be standard of care.

A total of 43% of patients newly diagnosed with cancer report fatigue before even starting their conventional anti-cancer therapies [47], and fatigue is a bidirectional predictor of poor lifestyle choices and willingness to engage with exercise or nutritional interventions [48,49,50,51]. The clinical evidence suggests that unresolved pre-treatment fatigue predicts a >3-fold risk of having more severe post-treatment fatigue [52,53], yet fatigue due to a vitamin D deficiency is one of the easiest causes of fatigue to treat [44,45,46]. One of the mechanisms of fatigue amelioration from vitamin D is enhanced skeletal muscle mitochondrial function [54,55,56]. Of note, many patients who were treated for vitamin D deficiency in our cohort only recognized their chronic fatigue symptoms after experiencing the energy improvement with their replacement therapy, as many had adapted to the deficient state for decades.

Lastly, vitamin D sufficiency likely has a protective effect on some of the toxicities associated with chemotherapy, such as paclitaxel-associated neuropathy [57,58], risk of diabetes [59,60] and impaired cognitive function [61,62,63]. Vitamin D replacement therapy is extremely well tolerated, with most experts agreeing that risk of hypercalcemia in healthy children and adults is negligible with D25 levels < 100 ng/mL [64,65,66].

Given the current state of clinical evidence, the biological plausibility of vitamin D having anti-cancer effects in ER- and HER2+ breast cancer, and the convincing evidence base that it promotes wellness and improves quality of life, it should be standard of care, until RCT evidence suggests otherwise, to screen for and treat a vitamin D deficiency in all patients with breast cancer, particularly the ER- and HER2+ subtypes. That said, prospective implementation studies are needed to verify risk/benefit in the various breast cancer subtypes, and in the context of a stressful diagnosis of breast cancer, query whether fatigue can significantly be improved with vitamin D replacement therapy and lead to better odds of healthy lifestyle changes.

## Figures and Tables

**Table 1:** Baseline demographics and characteristics.

Variables	*N* (%)
**Baseline Vitamin D**	
<30 ng/mL	129 (65.8)
≥30 ng/mL	67 (34.2)
**Age at diagnosis**	
<50 years	101 (51.5)
≥50 years	95 (48.5)
**BMI**	
<30 kg/m^2^	108 (55.1)
≥30 kg/m^2^	88 (44.9)
**Race**	
White	130 (66.3)
African American	42 (21.4)
Hispanic	15 (7.7)
Others	9 (4.6)
**Tumor size**	
<2 cm	123 (64.1)
≥2 cm	69 (35.9)
Unknown	4
**Number of metastatic lymph nodes**	
0	108 (55.7)
≥1	86 (44.3)
Unknown	2
**Lymphovascular invasion**	
No	104 (63.8)
Yes	59 (36.2)
Unknown	33
**ER status**	
Negative	56 (29.5)
Positive	134 (70.5)
Unknown	6
**PR status**	
Negative	89 (46.6)
Positive	102 (53.4)
Unknown	5
**Type of chemotherapy**	
Neoadjuvant	97 (49.5)
Adjuvant	99 (50.5)
**Definitive Surgery**	
Lumpectomy	84 (43.1)
Mastectomy	111 (56.9)
Unknown	1
**Radiation therapy**	
No	52 (26.5)
Yes	144 (73.5)
**Pertuzumab**	
No	98 (50)
Yes	98 (50)
**Disease progression**	
No	165 (84.2)
Yes	31 (15.8)
**Vitamin D response after supplementation**	
Responders	76 (60.3)
low	27
medium	31
high	18
Non-responders	50 (39.7)
Unknown	3

**Table 2:** Univariate Cox proportional hazard analysis (* reflects statistical significance achieved).

Variables	Hazard Ratio	95% CI	*p*-Value
**Vitamin D response after supplementation**			
Responders (reference)			
Non-responders	1.7	0.71–4.3	0.23
**Vitamin D response after supplementation**			
High responders (reference)			
Medium responders	1.4	0.26–7.8	0.69
Low responders	1.1	0.19–6.8	0.89
Non-responders	2.1	0.46–9.6	0.34
**Baseline Vitamin D**			
>=30 ng/mL (reference)			
<30 ng/mL	0.76	0.37–1.6	0.45
**Age at diagnosis**			
<50 years (reference)			
>=50 years	1.1	0.56–2.3	0.73
**BMI**			
<30 kg/m^2^ (reference)			
>=30 kg/m^2^	0.41	0.18–0.91	0.03 *
**Race**			
White (reference)			
Others	1.3	0.62–2.6	0.50
**Tumor size**			
<2 cm (reference)			
>=2 cm	1.3	0.64–2.8	0.44
**Number of metastatic lymph nodes**			
0 (reference)			
>=1	1.4	0.69–2.9	0.33
**Lymphovascular invasion**			
No (reference)			
Yes	2.3	1.03–5.3	0.04 *
**ER status**			
Negative (reference)			
Positive	0.79	0.37–1.7	0.53
**PR status**			
Negative (reference)			
Positive	0.81	0.40–1.7	0.57
**Type of chemotherapy**			
Adjuvant (reference)			
Neoadjuvant	3.8	1.7–8.7	0.001 *
**Definitive Surgery**			
Lumpectomy (reference)			
Mastectomy	1.5	0.72–3.2	0.28
**Radiation therapy**			
No (reference)			
Yes	1.4	0.58–3.5	0.44
**Pertuzumab**			
No (reference)			
Yes	2.5	1.2–5.5	0.02 *
**Nuclear grade**			
1 or 2 (reference)			
3	1.4	0.59–3.5	0.43

**Table 3:** Multivariate Cox regression analysis (* reflects statistical significance achieved).

Variables	Hazard Ratio	95% CI	*p*-Value
**BMI**			
<30 kg/m^2^ (reference)			
≥30 kg/m^2^	0.46	0.18–1.1	0.09
**Lymphovascular invasion**			
No (reference)			
Yes	2.3	1.01–5.4	0.05 *
**Type of chemotherapy**			
Adjuvant (reference)			
Neoadjuvant	4.0	1.4–11.3	0.008 *
**Pertuzumab**			
No (reference)			
Yes	1.2	0.45–3.4	0.07

## Data Availability

De-identified data is stored in two separate locations with access currently limited to current City of Hope Chicago (formerly CTCA MRMC) staff and faculty.

## Abbreviations

ER	estrogen receptor
PR	progesterone receptor
HER2	human epidermal growth factor receptor 2
D25	serum 25-hydroxyvitamin D
RCTs	randomized controlled trials
pCR	pathological complete response
DFS	disease-free survival
CTCA MRMC	Cancer Treatment Centers of America Midwestern Regional Medical Center
IRB	institutional review board
BMI	body mass index
HR	hazard ratio
CI	confidence interval
NK	natural killer
VDR	vitamin D receptor
IHC	immunohistochemistry
FISH	fluorescent in situ hybridization
VDBP	vitamin D-binding protein
